# Trade-Off Analysis to Determine Environmental Flows in a Highly Regulated Watershed

**DOI:** 10.1038/s41598-018-32126-6

**Published:** 2018-09-20

**Authors:** Aiping Pang, Chunhui Li, Tao Sun, Wei Yang, Zhifeng Yang

**Affiliations:** 1 State Key Laboratory of Water Environment Simulation, School of Environment, Beijing Normal University, Beijing, 100875 China; 2 Department of Public Management, Party School of C.P.C. Nanjing Committee, Nanjing, 210046 China

## Abstract

In this study, we proposed an approach to recommend environmental flows in highly regulated areas, considering the multiple time scales of hydrological processes and water requirements. Water resources were seasonally allocated to the agricultural sector using a benefit-maximizing model, based on water deficiency at different crop growth stages. The economic feedback was evaluated after securing different levels of initial environmental flows. The final environmental flows were recommended to maintain a balance between the ecosystem and irrigation water needs. A case study was applied in the Baiyangdian watershed, China. The results show that a benefit-maximizing model can reduce the total economic losses to the maximum potential, which contributes to alleviating water use conflicts between agriculture and the ecosystem. However, the environmental flows cannot be maintained without the sacrifice of production losses, except for in extremely wet years. Average environmental flows could be secured at around 3.1, 4.3 and 5.4 × 10^8^ m^3^ in dry, average, and wet years, respectively, with less than 10% production loss. Additional water transfer projects, as well as economic compensation strategies, are suggested to meet both ecosystem and agricultural needs. The planned economic compensation during 2010–2015 was 16.3 × 10^8^ Yuan, giving priority to securing environmental flows, and accounting for 7% of the total agricultural output value. The suggested amount of water resource transferred by the South-to-North Transfer Projects was 19 × 10^8^ m^3^, which is enough to alleviate water use conflicts between different stakeholders in dry years. This study provided a method to protect ecosystems in a more sustainable way.

## Introduction

Large amounts of water are diverted for irrigation processes and other human activities in many river basins worldwide^1^. Approximately 70% of natural water resources are extracted annually from river systems to supply agricultural irrigation^2,3^. The continued decline in water availability has a disproportionate impact on freshwater habitats, particularly riparian floodplains, wetland, and estuaries^4^. As the population grows worldwide, we will become even more dependent on irrigation processes to ensure sufficient food supplies. Conflicting demands between water extracted for irrigation and water required to meet environmental flow needs has therefor become a key issue in sustainable ecological protection and socioeconomic development^1,5^.

Environmental flow assessment, which is defined as the amount of water needed in a given ecosystem, has become an important tool in ecosystem restoration and water resource management^4,6,7^. In general, hydrological, hydraulic, habitat, and holistic methods have been used for designing environmental flows with the primary objective of ecosystem protection^8^. However, water use stakeholders, such as those involved in agriculture, often have difficulty in accepting water requirements for the ecosystem defined on the basis of ecological objectives, because of possible economic losses caused by the satisfaction of environmental flows under limited water resource conditions.

Balancing the outcomes of water utilization between ecosystems and human uses has become an important issue to be addressed when carrying our environmental flow allocations and managing water resources^3,9–13^. An increasing body of literature documents the development of optimization models that could be used to improve water allocation decisions^14–16^. Cai (2004) used scenario analysis to evaluate the trade-offs between irrigation water use and ecological water use, with the background of the South-to-North Transfer Project^3^. McCartney *et al*.^7^ stressed the necessity of integrating ecological economics into environmental water decisions^7^. The Water Evaluation and Planning System Version 21 (WEAP21) was previously integrated with a water resource management process to analyze water allocation-related issues through a scenario-based approach^17^. Similarly, in a case study of the Murray-Darling Basin in Australia, the economic benefits of allocating water to irrigation were analysed using a farm production model based on an irrigation survey data set^18^. Pang *et al*.^18^ developed a conflict analysis framework that could be used to determine the recommended environmental flows after balancing water requirements for ecosystem protection and irrigation processes^19^. Furthermore, some researchers have tried to optimize decisions about water allocation by using a probabilistic graphical model^11,20^.

Attempts to quantify agricultural losses related to ecosystem protections have focused mainly on assessing the outcomes of water allocation plans under alternative agricultural and environmental policy scenarios^21^, or on analysing differences in yield losses among crops^19^. However, the conflicting nature of water use means that one objective cannot be improved without degrading one or more other objectives, especially in arid and semi-arid areas^22^. In semi-arid areas, in particular, these conflicts seem to be irreconcilable contradictions. Reducing the economic losses caused by the maintenance of environmental flows, by considering the seasonality of hydrology conditions and water requirements, has received little attention so far. Exploring these factors could help us to provide the best solutions for managing environmental flows.

**Figure 1:**
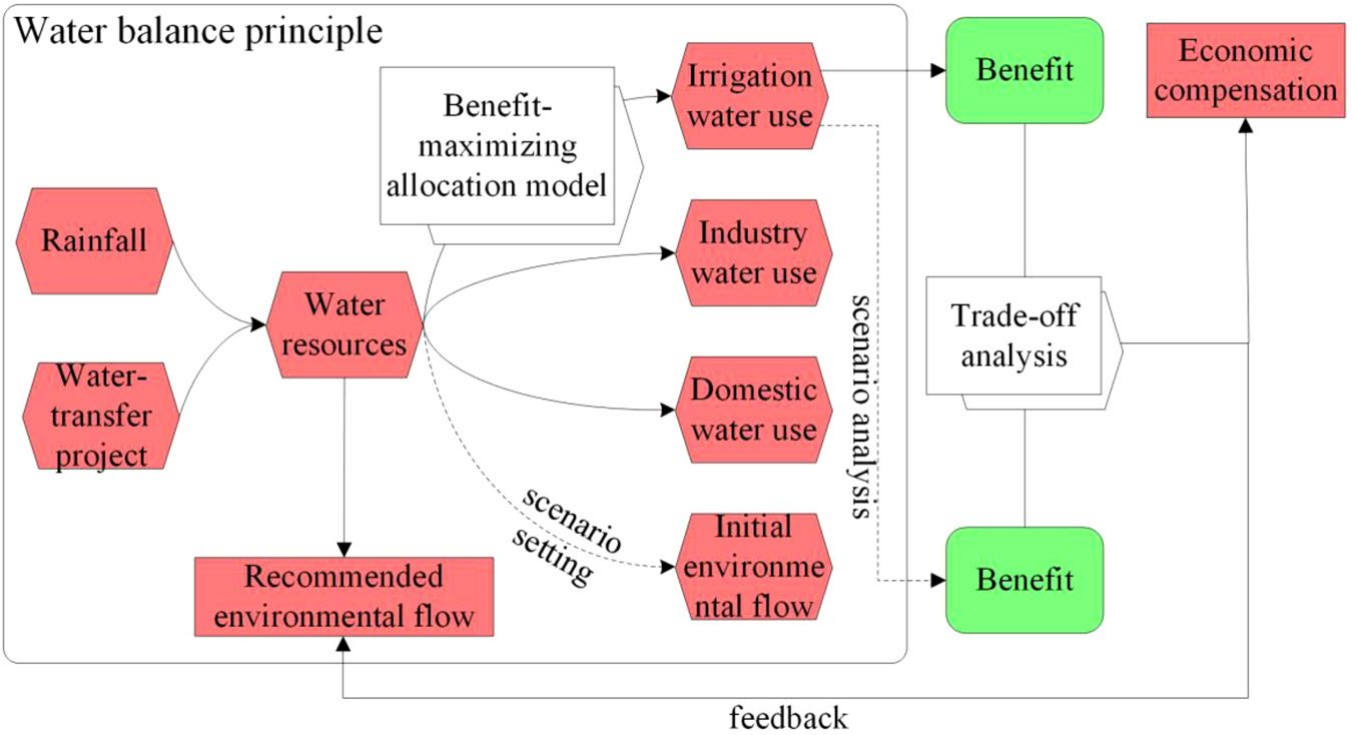
Hierarchical structure of the integrated model.

In this study, we developed an integrated approach to recommend what environmental flows should be utilized, by evaluating water resource allocation outcomes influenced by hydrological processes on multi-time scales (Fig. 1). A benefit-maximizing model was applied to the water allocation process. The environmental flow levels were recommended by the different degrees of economic losses that would occur under different scenarios. The Baiyangdian watershed, in China, was used as the case study area, and the environmental flows were recommended based on the feedback derived from irrigation benefits after securing different levels of initial environmental flows (see Methods).

There is little hope of resolving the conflict between agricultural water demand and ecological water demand in arid and semi-arid areas^3^. The irrigation stakeholders have no motive to accept the economic losses caused by the maintenance of environmental flows under limited water resource conditions. To solve this problem, fair economic compensation could be offered in exchange for water flow changes^23,24^. Another way to solve water use conflicts between agriculture and the ecosystem is through water transfer projects^3^. Economic compensation to secure the desired environmental flows, and transferring water resources from outside the watershed, were also recommended.

## Results

### Irrigation Water Demand and Water Resources for Irrigation

**Figure 2:**
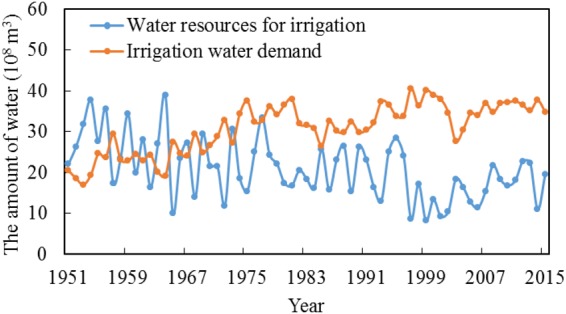
Irrigation water demand and water resources for irrigation.

Irrigation water demand has shown an increasing trend (Fig. 2) with the expansion of the crop planting area. The total planting area for crops was 0.47 million ha during 1951–1956, and this almost doubled from 2010–2015 (Fig. S1). Despite the high level of agricultural water demand, water resources that can be used for irrigation have been decreasing year by year (Fig. 2). The reasons for increasing water demand include the expansion of agriculture to produce more food (Fig. S1), the steady growth of the local population, and the geometric increase of gross industrial output (Fig. S2). However, the total water resources, which exhibit a decreasing trend (Fig. S3), cannot meet the demand of all water use stakeholders. Influenced by climate change, the average effective rainfall during the most recent ten years has decreased by almost 10% compared to that the first ten years, which results in more dependence on irrigation. Except in the years 1985, 1988, 1990 and 1995, which experienced abundant precipitation, the existing water resources could barely meet the irrigation water demand. Since 1978, the conflicts between irrigation water demand and limited water resources for irrigation have been intensifying. In 1997–2002, water resources for irrigation only accounted for 20–30% of the irrigation water demand. A severe shortage of water directly results in the reduction of crop production. The irrigation stakeholders were reluctant to give up water use rights to secure the most appropriate environmental flows.

### Benefits of Irrigation Processes

**Figure 3:**
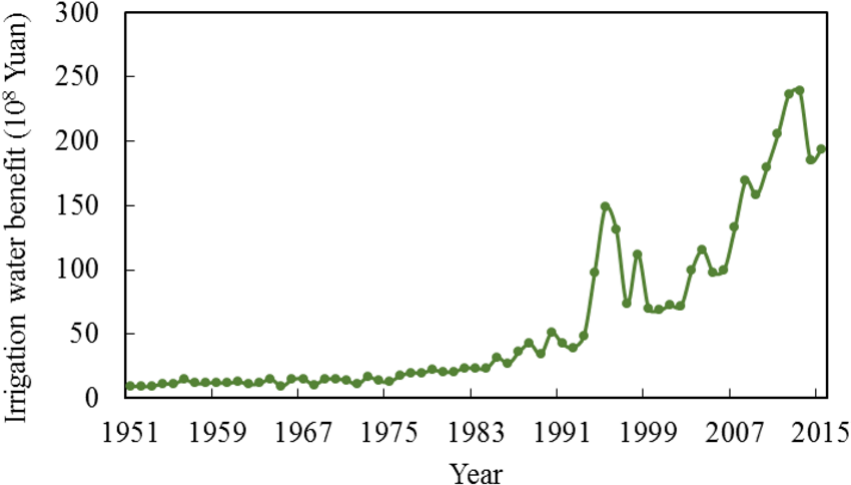
Benefits of irrigation processes.

The benefits of irrigation are influenced by the unit water value and the amount of water used for irrigation. Despite water resources for irrigation decreasing slightly during 1951–2015, the unit water value of irrigation has demonstrated geometric growth since 1978 (Fig. S4). The application of chemical fertilizer greatly improved the production of crops, which in turn positively influenced the unit water value. The geometric trend of the unit water value after 1978 was the direct result of the benefit of irrigation undergoing a similar trend (Fig. 3). Before 1978, the average benefit for irrigation was 13 × 10^8^ Yuan, which doubled after only ten years. From 2010–2015, the average irrigation benefit reached 207 × 10^8^ Yuan, which is almost 16 times higher than the average benefit before 1978. The financial benefits of irrigation reached as high as 148.8 × 10^8^ Yuan, almost double that of later years. This peak could be explained by the boom in the crop price for winter wheat and summer corn during 1994–1998, which made irrigated water more valuable.

### Agricultural Losses Due to Ensuring Environmental Flows

**Figure 4:**
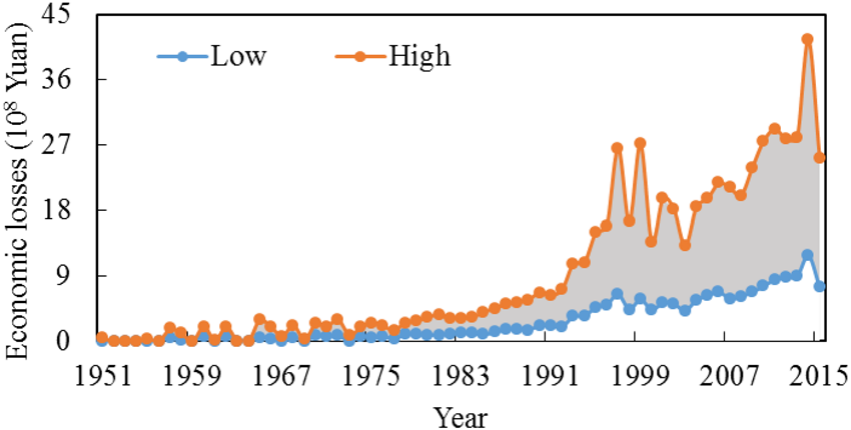
Agricultural economic losses for ensuring initial environmental flows.

The economic loss after ensuring the initial environmental flows was 3–14% of the irrigation benefit. In a highly regulated watershed, water resources can be reserved by the reservoirs and dams, or in the form of ground water. The benefit-maximizing allocation model can seasonally allocate water resources to the key crop growth stages, to reduce the total economic losses caused by maintaining environmental flows. In the Baiyangdian watershed, except in extremely wet years, losses to irrigation indeed existed due to ecological protections. After 1993 especially, the agricultural losses caused by the maintenance of environmental flows was 10–30 × 10^8^ Yuan (Fig. 4), which was beyond the range that was acceptable to irrigation stakeholders. The agricultural losses should be controlled in a more flexible way, such as by lowering the environmental flow standard in dry years.

### Recommended Environmental Flows

**Figure 5:**
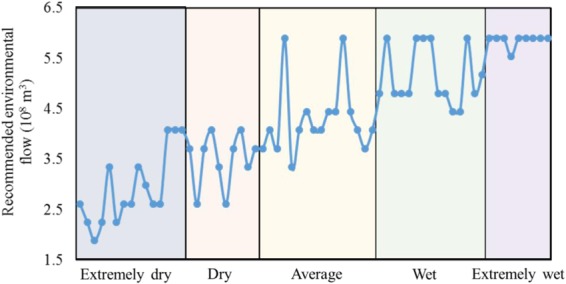
Recommended environmental flows under different hydrological year.

In general, ecosystems can obtain more water in wet years, and can maintain their basic ecological functions in dry years, after trade-offs are made with irrigation water use. In dry years, the agricultural losses were beyond the acceptable range for irrigation stakeholders and local government after maintaining a higher level of environmental flow. Therefore, in dry hydrological years, the recommended environmental flow for the Baiyangdian Lake was always under 4 × 10^8^ m^3^ (Fig. 5). In extremely wet years, the average irrigation water use was higher than the irrigation water requirement—the amount of water resource available for irrigation and high-level environmental flow (5.9 × 10^8^ m^3^) to occur without causing any damage. In most of the average years and wet years, the recommended environmental flows were around 4.5 × 10^8^ m^3^. Exceptions occurred in years with abundant rainfall in the months with a high unit water value; in other words, when little water use conflict existed between agriculture and the ecosystem. In a typical, average year such as 1951, the annual water resources only accounted for 60% of that of a typical extremely wet year such as 1964. However, in February—the time of the key crop growth stage—water resources used in 1951 were 1.7 times that used in 1964. Thus, it is better to allocate the same level of environmental flow in each of these years.

## Discussion

Water resources for irrigation should be allocated in the most beneficial way, starting with the highest unit values for the water volume applied to the various crop growth stages, followed by successively decreasing water values. When giving priority to crop growth stages with high water unit values, fulfilling the demand with plenty of effective rainfall at different crop growth stages may result in less damage by freeing up environmental flow. The method of considering multiple time scales could guarantee environmental flows are maintained in a healthy manner (within the boundary of initially low and high levels of environmental flows), and could reduce the production losses to the maximum potential. In the following section, we discuss the impartiality of the priority of securing industrial and domestic water use. The methods for solving this issue discussed in order to provide a basis for water resource managers include: (1) how much money should be paid to irrigation stakeholders; and (2) how many water resources should be transferred to guarantee the sustainability of the economy and ecosystem in the Baiyangdian watershed.

### Water Use and Water Use Benefits of Different Stakeholders

**Figure 6:**
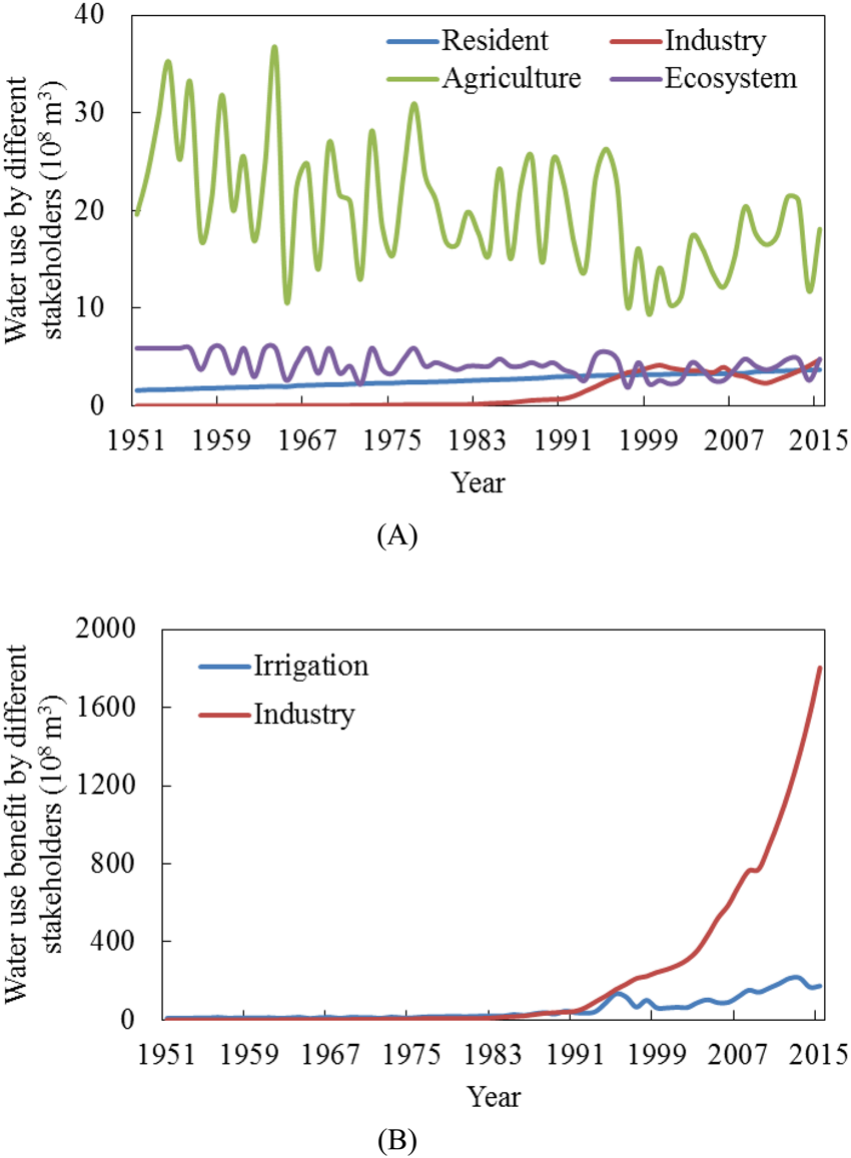
Water resources allocation and the water use benefit for different stakeholders.

It should be noted that environmental flow insurance is not the only cause of agricultural water use deficiency; it can also be caused by water resources being used for industrial and domestic purposes. The framework we developed here primarily considers the changes in benefits derived from irrigation and ecological uses, caused by the maintenance of environmental flows. It is essential to illustrate the share of water allocation by each stakeholder and the values the water resources could produce. In the Baiyangdian watershed, agriculture is the primary water use sector. According to the Baoding Water Conservancy Bureau^25^, the average irrigation water use during 1985–2010 accounted for 81% of the total water resources used. Despite reallocation of water resources to maintain environmental flows under different hydrological conditions, the average irrigation water use still accounts for 70% of the total water resources use (Fig. 6A).

Domestic water use has increased predictably along with the increase in the population. Industry water use, however, has increased exponentially from 1990–2000, with an annual growth rate as high as 600%. The government of Baoding City, which shares 70% of the Baiyandian watershed, established a series of industry water saving measures during the ninth five-year plan period (2001–2005). The government has established a water consumption quota for industry water use stakeholders, according to their water consumption situation and water-saving potential, and imposed fines of 1–16 times to those who used more than 20%–80% of their water consumption quota. Meanwhile, the local government has also invested one billion RMB per year to support enterprises that promote cooling water cycle technology. Since 2000, water use by industry has stabilized at 3.4 × 10^8^ m^3^.

The water value is measurable in both the agricultural and industrial areas. The water values produced by these two areas started to increase after 1978, when China began to carry out the reform and opening-up policy (Fig. 6B). The water values showed more rapid growth in industry than in irrigation. The diversity between these two departments has increased since then, especially in 2015 when the water value produced by industry was tenfold than that of the agricultural sector, with less than a third of the allocated water resources. Unlike in the agricultural sector, the industries in the Baiyangdian Watershed have little water-saving potential. The amount of water resource used by agriculture needs to reduce by 10% to meet the needs of the ecosystem and other stakeholders. Currently, there is no effective way to calculate the water values for the ecosystem, but water use for the ecosystem is very important and requires attention. In the Baiyangdian watershed, water use for industry and households usually occurs in a planned manner, and often there is little water-saving potential. It is reasonable to secure environmental flows by reallocating water resources in the agricultural sector.

### Economic Compensation to Guarantee Environmental Flows

**Figure 7:**
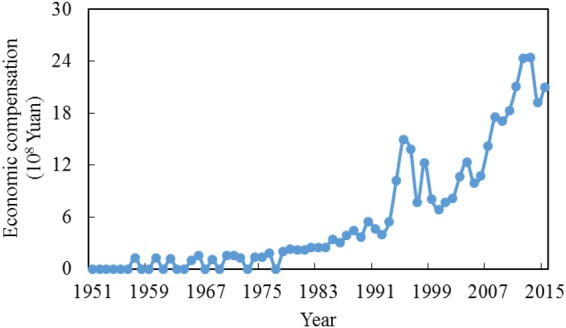
Economic compensation for different hydrological years.

The conflicting nature of water use mean that one objective cannot be improved without degrading one or more other objectives^22^. The benefit of maximizing trade-offs could relief the water use conflicts between agriculture and the ecosystem, thus reducing the losses to the maximum potential. However, the economic losses do indeed exist, thus irrigation stakeholders unlikely to want to give up water use rights. An economic compensation could be an effective way to alleviate this conflict. Economic compensation could be determined based on the economic losses after maintaining a certain level of environmental flow (Fig. 7).

Economic compensation tends to increase for two main reasons: growing competition for water resources, and increasing water use values (Fig. S4). To secure the same level of environmental flow, irrigation has endured more economic losses in recent years than in the years prior to this, with the same degree of production loss. The average economic compensation from 2010–2015 were 16.3 × 10^8^ Yuan, which is almost 41 times higher than the compensation offered from 1951–1960. Given the scarcity of water resources and the increase of the grain price, maintaining the security of environmental flows will cause more monetary losses in the future. It should be noted that the reallocation of water resources to maintain environmental flows for the Baiyangdan Lake started from the premise that reservoirs or dams are under normal functional regulation, and the groundwater can also be maintained at a normal level. In the Baiyangdian watershed, however, farmers are more dependent on the ground water to secure their harvest. The average exploitation of ground water accounted for 90% of the total water resources in Baoding city, 82% of which was used by irrigation. The overuse of ground water has caused a series of environmental issues, such as the ground water level dropping, ground funnelling and land subsidence. Economic compensation can be given to farmers only under the condition that the ground water was exploited in reasonable and planned manner.

### Water Transfer Project

**Figure 8:**
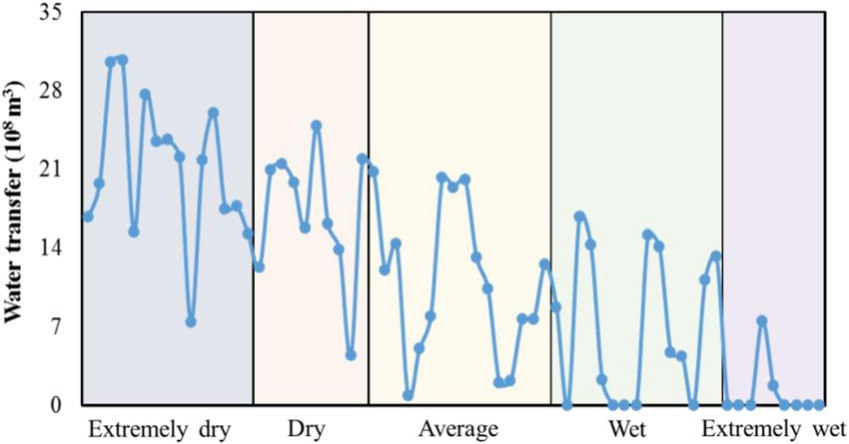
The amount of water transferred under different hydrological years.

We assume that if the water resources are allocated to the ecosystem as the priority, it may cause enormous pressure on the agricultural sector. Economic compensation for up to 10% of the economic losses may not thoroughly solve water use conflicts between irrigation and ecological protection. The South-to-North Water Transfer Project started in 2002, and planned the transfer of 448 × 10^8^ m^3^ of water to solve the water deficiency problem in the north area of China. The Baiyangdian watershed is also on the list for this transfer project. Figure 8 illustrates the amount of water resource that should be transferred from outside of the watershed to fulfil the water requirements of all the local stakeholders. Generally, more water resources should be transferred in relatively dry years, while transfers of water are barely required in wet years. The suggested amount of water resource transferred by the South-to-North Transfer Project in extremely dry, dry, average, wet, and extremely dry years were 21, 17, 11, 7 and 1 × 10^8^ m^3^, respectively. Exceptions will occur frequently due to the seasonality of precipitation and irrigation water requirements. As an example, in 1995, an extremely wet year with an abundance of precipitation, the irrigation water requirement was 33.9 × 10^8^ m^3^, and in this case around 6.5 × 10^8^ m^3^ should be transferred to the watershed. In some dry years, the effective rainfall could meet the agricultural water demand, thus little in the way of water resources would be needed for irrigation purposes and then the water transfer project is less essential.

### Uncertainties and Further Model Development

Due to the complicated relationships among hydrological processes, human activities, and ecological processes, various uncertainties exist in the environmental flow determination process.

Our study primarily conducted trade-offs between agriculture and the ecosystem, because the agricultural sector in the Baiyangdian watershed consumes 76% of the water resources^26^. Our approach could be applicable in areas/basins with similar conditions, such as the Yellow River basin in China^3,24^, the Conchos River basin in Mexico^23^, the Nile River basin in Africa^27^, and the Murray-Darling basin in Australia^21^. However, in areas with high industrial and domestic water demand, water saving potential and the quantity of return flows should not be neglected.

In our research, we use the precipitation and water production rate to calculate the total water resource, which includes both surface and ground water. However, the extraction of surface water and ground water may result in different outcomes. The relationship between surface water and groundwater in the hydrological cycle is an important issue that should be addressed in future analyses of agricultural water shortages. The irrigation stakeholders were more reluctant to give up water use rights to secure environmental flows. The average precipitation during the periods of 1951–1960 and 2001–2010 decreased by 23%, while evapotranspiration stayed at a similar level. Due to the effects of climate change, drought is expected to worsen in various regions in the future^28^. In addition, there has a warming trend predicted from 2006–2099 in China, and the northern regions (including the Baiyangdian watershed) are expected to experience more warming^29^. In the future, the Baiyangdian watershed will face pressure from both droughts and warming, and the water use conflicts between agriculture and the ecosystem will be intensified. The goal of our study was to propose an assessment framework to provide support for management decisions, especially in highly regulated areas. The developed model is broadly generalizable and allows for the incorporation of additional factors into the analyses, such as the effects of global climate change and increased human activities on water availability and demand. Our research should be taken as a platform for further negotiations between stakeholders in terms of water quantity and economic outcomes instead of providing an optimized water allocation model for ecosystems and human activities.

## Methods

We proposed an integrated model for recommending environmental flows in a highly regulated watershed with limited water resources and severe water use conflicts, which consists of a water resource allocation system with a benefit-maximizing pattern for agricultural water allocation, and a trade-off analysis model with a feedback system for the recommended environmental flows.

### Water Resources Allocation Model

In the highly regulated area of the Baiyangdian watershed, the river flow regime has been highly manipulated through human activities. The water resources produced by precipitation and transferred from outside of the watershed are stored in lakes, reservoirs and the groundwater, which will eventually be extracted by different stakeholders. Annual water resources used for irrigation purposes, after fulfilling the industrial, household, and initial environmental flow water requirements could be calculated based on the water balance principle:

1 $${{\rm{W}}}_{{\rm{local}}}+{{\rm{W}}}_{{\rm{t}}{\rm{r}}{\rm{a}}{\rm{n}}{\rm{s}}{\rm{f}}{\rm{e}}{\rm{r}}}={{\rm{W}}}_{{\rm{ir}}}^{{\rm{u}}}+{{\rm{W}}}_{{\rm{in}}}+{{\rm{W}}}_{{\rm{d}}}+{{\rm{W}}}_{{\rm{e}}}+{{\rm{W}}}_{{\rm{out}}}$$

where W_local_ is the total water resources produced within the watershed (m^3^); W_transfer_ is the water resources transferred from outside of the watershed (m^3^); ${{\rm{W}}}_{{\rm{ir}}}^{{\rm{u}}}$ is irrigation water use (m^3^); W_in_ is industrial water use (m^3^); W_d_ is domestic water use (m^3^); W_e_ is the initial environmental flow for ecosystems (m^3^) (usually determined based on the objective of ecosystem protection); and W_out_ is the water resources flow out of the watershed.

It is difficult to obtain the long term and seasonal scale of ground water data, therefore we introduced the concept of the water production rate, which is the ratio of total water resources and precipitation^30,31^. The total water resources W_local_ can be calculated by the following equation:

2 $${{\rm{W}}}_{{\rm{local}}}={{\rm{\alpha }}S}_{{\rm{T}}}{{\rm{P}}}_{{\rm{tr}}}$$

where α is the water production rate (dimensionless); S_T_ is the watershed area (m^2^); and P_tr_ is the total rainfall (m).

The initial environmental flow is usually determined based on the typical ecological objectives for environmental protection. Sun *et al*.^6^ developed a method for quantifying environmental flows, while integrating multiple ecological objectives^6^:

3 $${{\rm{W}}}_{{\rm{e}}}={\sum }_{{\rm{i}}=1}^{{\rm{n}}}{{\rm{W}}}_{{\rm{i}}}+\,{\rm{\max }}{(W}_{{\rm{j}}1},{{\rm{W}}}_{{\rm{j}}2},\mathrm{...}{,{\rm{W}}}_{{\rm{jm}}})$$

where W_e_ is the initial environmental flow (m^3^); max(a,b) denotes the maximum value for variables a and b, W_i_ is the consumptive water volume (m^3^); W_j_ is the non-consumptive water volume (m^3^); and n and m indicate the number of objectives for consumptive and non-consumptive water volumes, respectively. The rule of summation is used to calculate the consumptive water requirements, and the rule of compatibility (i.e., maximum principle) is adopted to estimate the non-consumptive requirements.

### Benefit-Maximizing Allocation Model

Different crop growth stages require different amounts of water, and can consequently produce different water values. Water shortages in different stages can therefore result in different degrees of production damage. Seasonally, we propose a benefit-maximizing pattern for agricultural water resource allocation. The crop growth stage with the highest unit water value is given the priority to obtain water resources, followed by crop growth stages with successively decreasing water values (the order is represented as 1^th^, 2^th^…n^th^).

The unit water value for crop growth stage j^th^ (1 ≤ j ≤ n) can be calculated as follows:

4 $${{\rm{UWV}}}_{{\rm{j}}}={{\rm{Qq}}}_{{\rm{m}}}{({{\rm{k}}}_{{\rm{y}}})}_{{\rm{j}}}{\rm{S}}\frac{{\rm{1}}}{{{\rm{W}}}_{{\rm{a}}}}$$

where UWV is the unit water value, or the value produced by unit of water resource (Yuan/m^3^); Q is the crop price during the research period (Yuan/kg); q_m_ is the maximum potential crop yield (kg/ha); (k_y_)_j_ is the yield response factor in crop growth stage j^th^ (dimensionless); S is the planting area (ha); and W_a_ is the annual agricultural water demand (m^3^).

Water resources for crop growth stage j^th^ (1 ≤ j ≤ n) can be allocated as follows:

5 $${({{\rm{W}}}_{{\rm{ir}}}^{{\rm{u}}})}_{{\rm{j}}}=\{{{\rm{W}}}_{{\rm{ir}}}^{{\rm{u}}}-\sum \begin{array}{c}0\\ {({{\rm{W}}}_{{\rm{ir}}}^{{\rm{r}}})}_{{\rm{j}}-1}\\ {({{\rm{W}}}_{{\rm{ir}}}^{{\rm{r}}})}_{{\rm{j}}}\end{array}\,\,\sum \begin{array}{ccc}{{\rm{W}}}_{{\rm{ir}}}^{{\rm{u}}} & \le  & \sum {{({\rm{W}}}_{{\rm{ir}}}^{{\rm{r}}})}_{{\rm{j}}-1}\\ {{({\rm{W}}}_{{\rm{ir}}}^{{\rm{r}}})}_{{\rm{j}}-1} & \le  & {{\rm{W}}}_{{\rm{ir}}}^{{\rm{u}}}\le \sum {{({\rm{W}}}_{{\rm{ir}}}^{{\rm{r}}})}_{{\rm{j}}}\\ {{\rm{W}}}_{{\rm{ir}}}^{{\rm{u}}} & \ge  & \sum {{({\rm{W}}}_{{\rm{ir}}}^{{\rm{r}}})}_{{\rm{j}}}\end{array}$$

where ${{\rm{W}}}_{{\rm{ir}}}^{{\rm{u}}}$ is the volume of water resource required for all growth stages (m^3^); ${{({\rm{W}}}_{{\rm{ir}}}^{{\rm{u}}})}_{{\rm{j}}}$ is the volume of water resource required for growth stage j^th^ (m^3^); and ${{({\rm{W}}}_{{\rm{ir}}}^{{\rm{r}}})}_{{\rm{j}}-{\rm{1}}}$ and ${{({\rm{W}}}_{{\rm{ir}}}^{{\rm{r}}})}_{{\rm{j}}}$ are the irrigation water requirements of growth stages (j − 1)^th^ and j^th^, respectively, (m^3^).

The irrigation water requirement ${{\rm{W}}}_{{\rm{ir}}}^{{\rm{r}}}$ is calculated from the agricultural water demand and effective rainfall:

6 $${{({\rm{W}}}_{{\rm{ir}}}^{{\rm{r}}})}_{{\rm{j}}}={{({\rm{W}}}_{{\rm{a}}})}_{{\rm{j}}}-{{({\rm{P}}}_{{\rm{er}}})}_{{\rm{j}}}{\rm{S}}$$

where (W_a_)_j_ is the agricultural water demand, or the total water demand by crops; (P_er_)_j_ is the effective rainfall (m); and S is the planting area (m^2^), all for crop growth stage j^th^. The agricultural water demand W_a_ can be determined based on the reference crop evapotranspiration and the planting area^24^:

7 $${{\rm{W}}}_{{\rm{a}}}={{\rm{ET}}}_{{\rm{m}}}{\rm{S}}$$

where ET_m_ is the reference crop evapotranspiration (m), which can be estimated based on the potential evapotranspiration (ET_0_) and crop coefficient (k_c_)^32^.

ET_0_ represents the evapotranspiration rate from a reference surface that is not short of water. We use the Penman-Monteith formulation^33^ to calculate this parameter:

8 $${{\rm{ET}}}_{{\rm{0}}}=\frac{\frac{{{\rm{p}}}_{{\rm{0}}}{\rm{\Delta }}}{{\rm{p}}{\rm{\gamma }}}+{\rm{0}}{\mathrm{.26}({\rm{E}}}_{{\rm{s}}}-{{\rm{E}}}_{{\rm{a}}})({1+\mathrm{cU}}_{{\rm{2}}})}{\frac{{{\rm{p}}}_{{\rm{0}}}{\rm{\Delta }}}{{\rm{p}}{\rm{\gamma }}}+1}$$

where p_0_ is the atmospheric pressure at sea level (kPa); p is the actual atmospheric pressure(kPa); E_s_ is the saturation vapor pressure (kPa); E_a_ is the actual vapor pressure (kPa); c is the correction coefficient of the wind speed (dimensionless); and U_2_ is the wind speed at height of 2 m (m/s); γ is psychrometric constant (kPa/°C); Δ is the rate of change of saturation specific humidity with air temperature (kPa/°C).

Effective rainfall is the portion of precipitation falling during the crop-growing period that infiltrates the soil surface and is available for plant consumptive use^34^. Effective rainfall does not include precipitation that is lost below the crop root zone, due to surface runoff, or because of soil surface evaporation. We estimated the effective rainfall following the USDA Soil Conservation Service^35^.

9 $${{\rm{P}}}_{{\rm{er}}}=\{\begin{array}{cc}{{\rm{P}}}_{{\rm{tr}}}(4.17-0.2{{\rm{P}}}_{{\rm{tr}}})/4.17 & {{\rm{P}}}_{{\rm{tr}}} < 0.0083\,{\rm{m}}/{\rm{day}}\\ 4.17+0.1{{\rm{P}}}_{{\rm{tr}}} & {{\rm{P}}}_{{\rm{tr}}}\ge 0.0083\,{\rm{m}}/{\rm{day}}\end{array}$$

where P_er_ is the effective rainfall (m), and P_tr_ is the total rainfall (m).

### Trade-Offs Between Agriculture and the Ecosystem

The objectives of the trade-off analysis were to maximize environmental flow for the ecosystem while minimizing production losses related to agricultural water shortages. Initial environmental flows from an ecosystem protection standpoint were taken as the boundaries of recommended environmental flows rather than as conclusive flow requirements^19^. We assume that low and high levels of initial environmental flows for ecosystem health can act as lower and upper boundaries. Scenario analysis was conducted to evaluate the irrigation benefits under different initial environmental flow settings (Fig. 1). For recommended environmental flows:

10 $${{\rm{W}}}_{{\rm{re}}}={\max ({\rm{W}}}_{{\rm{e}}})$$

Subject to:

11 $${\rm{L}}={{\rm{B}}}_{{\rm{a}}}-{{\rm{B}}}_{{\rm{ae}}}\le {\rm{X}}$$

12 $${{({\rm{W}}}_{{\rm{e}}})}_{\min }\le {{\rm{W}}}_{{\rm{re}}}\le {{({\rm{W}}}_{{\rm{e}}})}_{\max }$$

where (W_e_)_min_ and (W_e_)_max_ are the initial low and high environmental flows, respectively, based on the typical ecological objectives (m^3^); B_a_ and B_ae_ are the irrigation benefits before and after maintaining initial environmental flows (Yuan), respectively; L is the losses after maintaining different level of environmental flows (Yuan); and X is the upper limit of losses the local government can bear in order to maintain a healthy ecosystem.

The benefits of irrigation are the sum of the water values for different crop growth stages:

13 $${{\rm{B}}}_{{\rm{a}}}={\sum }_{{\rm{j}}}^{{\rm{n}}}{(\mathrm{UWV}\times {{\rm{W}}}_{{\rm{ir}}}^{{\rm{u}}})}_{{\rm{j}}}$$

Irrigation stakeholders operating under limited water resources are not willing to accept the economic losses caused by the reallocation of water resources. The economic losses (L) could be used as a compensation standard for irrigation stakeholders^24^.

### Site Description

**Figure 9:**
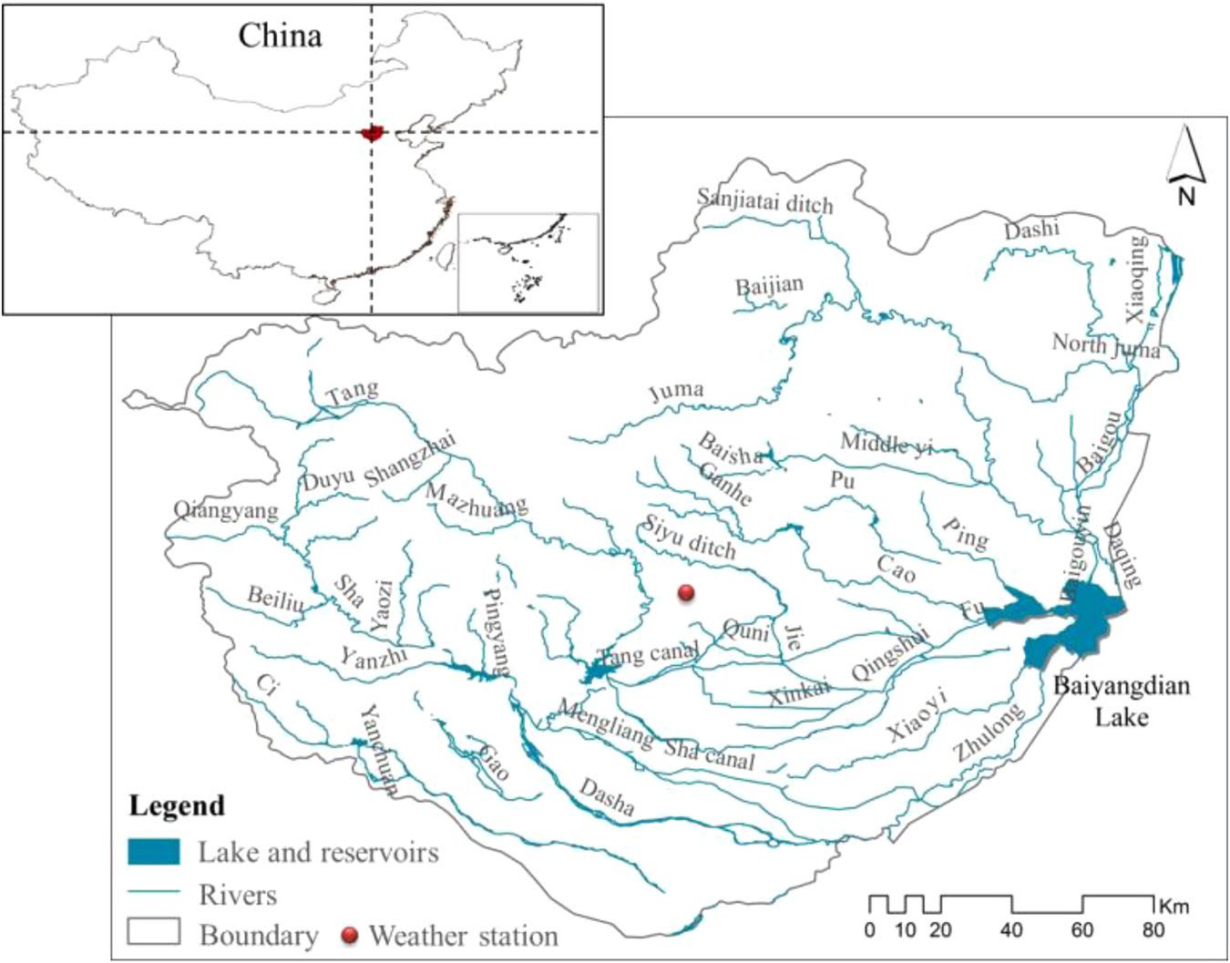
Location of the Baiyangdian watershed in North China.

Our approach was applied to the Baiyangdian watershed, which is located in the North China Plain (Fig. 9). The watershed extends in latitude from around 37.8° to 40.4°N and in longitude from around 113.3° to 116.6°E, with a total area of 31,200 km^2^. Agricultural activities are intensive in the watershed, with winter wheat and summer corn as the main crops, which are usually planted in a rotational way. The growth periods are October to May for winter wheat and June to September for summer corn. Both of the crops undergo four growth stages: the vegetative stage, flowering stage, yield formation stage, and ripening stage.

The river flow regime in the Baiyangdian watershed has been highly manipulated through human activities^36^. More than 100 large and small reservoirs have been established since1950s. The ground water has been seriously overdrawn, which has caused a regional cone of depression of deep groundwater in large area. In recent decades, water use for irrigation has accounted for 76% of the total water use, and water inflow into the Baiyangdian Lake has dramatically decreased^37^. The reduction of upper stream inflow has resulted in a decreasing water level and an increased frequency of the lake drying up—an event which occurred only once from 1919–1965 (in the spring of 1922)^26^, but which has occurred frequently since 1965. Recently, the lake has become a semi-closed water body with no natural outflows for periods of several years. There was barely any water flow into Baiyangdian Lake during the years of 1980–1987, 1965–1971 and 1997–2000; during the period of 1983–1987, Baiyangdian Lake was completely dried up.

### Data Source

The daily precipitation and the parameters to calculate the ET_0_ during the period of 1951–2015 were downloaded from the China meteorological data sharing service system^38^, supplemented by the global data sharing network^39^. To represent the actual local weather conditions, we used the average data of eight weather stations, which were located in or near the Baiyangdian watershed (Fig. S5).

Industrial and domestic water use data from 2000–2010 was derived from the Baoding water resources bulletin^40^, and the data for the years 1951–1999 and 2011–2015 was indirectly calculated by population and the gross value of industrial output (SI text 1), which was in turn derived from the Government Office of Hebei Province^41^ and the Baoding Bureau of Statistics^42^. Population and gross industrial output during 1951–2015 are illustrated in Fig. S2.

The water production rate used to calculate the total water resources is 0.18, which is the mean value according to the Water Resources Protection Bureau of the Haihe River basin^43^. The calculated total water resources are illustrated in Fig. S3. The planting area and production for winter wheat and summer corn during 1995–2015 were derived from the Hebei Rural Statistical Yearbook^44^. The activity data during 1951–1994 were indirectly calculated from the provincial scale, which came from the Sixty Years of New Hebei^45^. The calculation process can be seen in SI text 2 and the planting area and production for winter wheat and summer corn are illustrated in Fig. S4.

Crop yield response factors (k_y_) for five growth stages (vegetative period, flowering period, yield formation, ripening, and total growing period) of winter wheat and summer corn in the irrigation district were derived from Chen (1995)^46^, and are illustrated in Table 1. The maximum yields of winter wheat and summer corn are 6484 and 7184 kg/ha, respectively, which was taken from the maximum production data during 1951–2015. Crop prices during 1951–2015 were derived from the Chinese Agricultural Book^47^, a compilation of cost and benefit data of national agricultural products^48^, and the China Yearbook of Agricultural Price Survey^49^. In addition, the missing data were supplemented by a fitting equation.

**Table 1:** Crop yield response factors for winter wheat and summer corn in the irrigation district.

Growth stage	Winter wheat		Summer corn	
	Month	Crop yield response factors	Month	Crop yield response factors
Vegetative	Feb.–Mar.	0.2	June	0.4
Flowering	Apr.	0.6	July	1.5
Yield formation	May	0.5	Aug.	0.5
Ripening	—	—	Sept.	0.2
Total growth	Oct.–May	1.0	June–Sept.	1.25

According to Li (2008)^50^, the lowest and highest levels of environmental flow in the Baiyangdian Lake were estimated to be 1.89 × 10^8^ and 5.90 × 10^8^ m^3^, respectively, accounting for 6.7% and 20.1% of the total water resources. We assumed that the low and high levels of environmental flow that focused primarily on ecosystem health acted as the boundaries for the initial environmental flow. The flow were equally divided into ten security levels, accounting for 7.72%, 8.97%, 10.22%, 11.47%, 12.72%, 13.97%, 15.22%, 16.47%, 17.72%, and 18.97% of the total water supply in an average year. The trade-offs were conducted for twelve scenarios in which the economic benefits were evaluated after maintaining the low and high flows, together with the ten different security levels of environmental flows. To maintain a natural flow regime, temporal variations in natural river discharge were chosen as an indicator for the temporal variations in environmental flows^51^. The upper limit of losses (X) is 10% of the total benefits before securing environmental flows, as advised by local economic experts.

## Electronic supplementary material

Supplementry information
