# Supplementary material for: Trade-Off Analysis to Determine Environmental Flows in a Highly Regulated Watershed
Source: Sci Rep. 2018 Sep 20;8:14130. doi: 10.1038/s41598-018-32126-6 (PMC6148294; doi:10.1038/s41598-018-32126-6)
Supplement: Supplementary file 1 — Supplementry information [file 41598_2018_32126_MOESM1_ESM.pdf]

SI for

Trade off analysis to determine environmental flows in a highly regulated watershed

Aiping Pang [ab], Chunhui li [a] , Tao Sun [a], Wei Yang [a], Zhifeng Yang [a]

[a] *State Key Laboratory of Water Environment Simulation, School of Environment, Beijing Normal University, Beijing, 100875, China.*

[b] *Department of Public Management, Party School of C.P.C. Nanjing Committee , Nanjing 210023, China*

Text 1. For there is no detailed information for industrial and domestic water use during 1951-1999, and 2011-2015, we obtain that information by using the following equations:

$$(W_{in})_i = \frac{(W_{in})_j}{(G_{in})_j} \times (G_{in})_i$$

$$(W_d)_i = \frac{(W_d)_j}{(POP)_j} \times (POP)_i$$

where W_{in} and W_d are industrial and domestic water use, and G_{in} is the gross value of industrial output, POP is the total population, and i represents the year in which the data did not recorded by local government, while j represents the year in which the data could be obtained directly. In our case, i specifies the years 1951-1999 and 2011-2015, and j specifies the years 2000-2010.

Text 2. The planting area and production for winter wheat and summer corn during 1951-1994 was indirectly calculated from the provincial scale using the following equation.

$$(\mathrm{S_{watershed}})_\mathrm{i} = \frac{(\mathrm{S_{province}})_\mathrm{j}}{(\mathrm{S_{watershed}})_\mathrm{j}} \times (\mathrm{S_{province}})_\mathrm{i}$$

$$(\mathrm{P_{watershed}})_\mathrm{i} = (\mathrm{P_{province}})_\mathrm{i}$$

where $\mathrm{S_{watershed}}$ and $\mathrm{S_{province}}$ are the planting area in the watershed scale and province scale, $\mathrm{P_{watershed}}$ and $\mathrm{P_{province}}$ are the production in the watershed scale and province scale, i represents the year in which the data did not recorded by local government, while j represents the year in which the data could be obtained directly. In our case, i specify the years 1951-1994, and j specify the years 1995-2015.

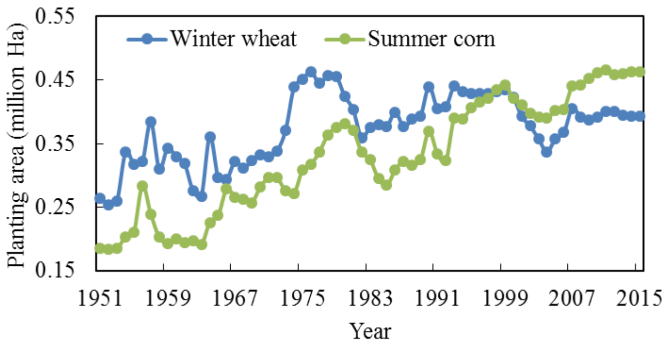

(A)

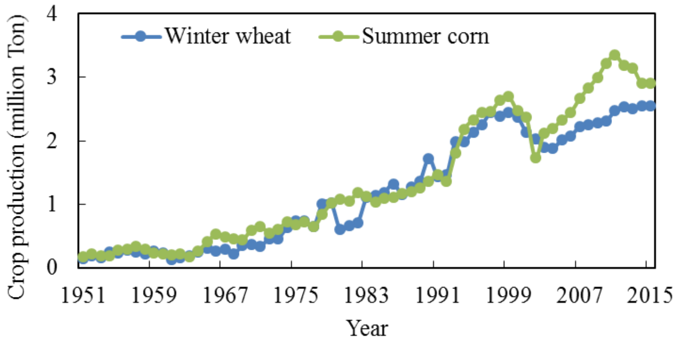

(B)

Figure S1 Planting information for winter wheat and summer corn, (A) represent planting area and (B) is production

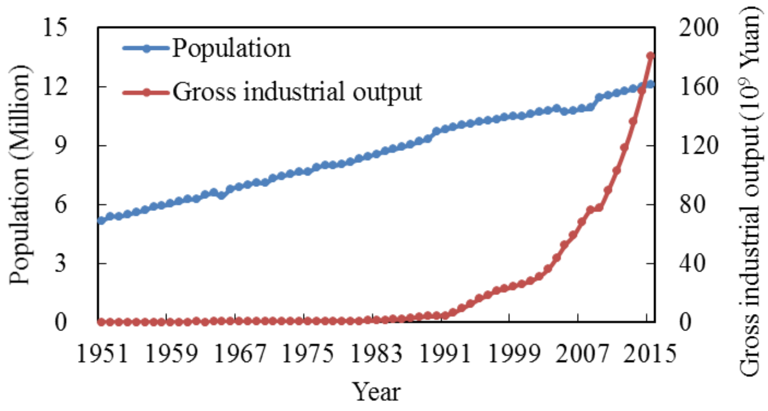

Figure S2 Population and gross industrial output during 1951-2015

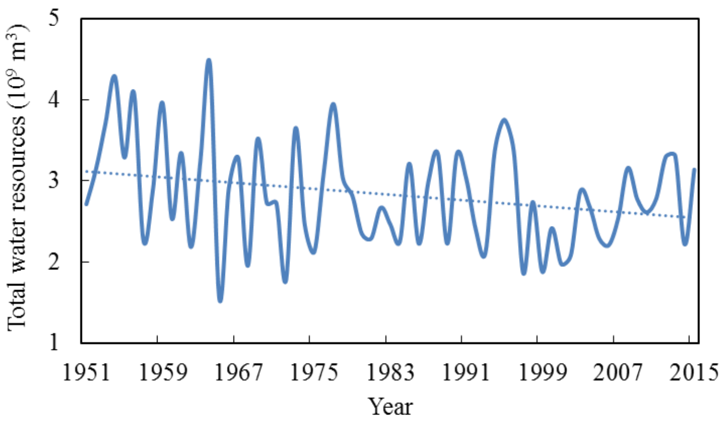

Figure S3 Total water resources during 1951-2015

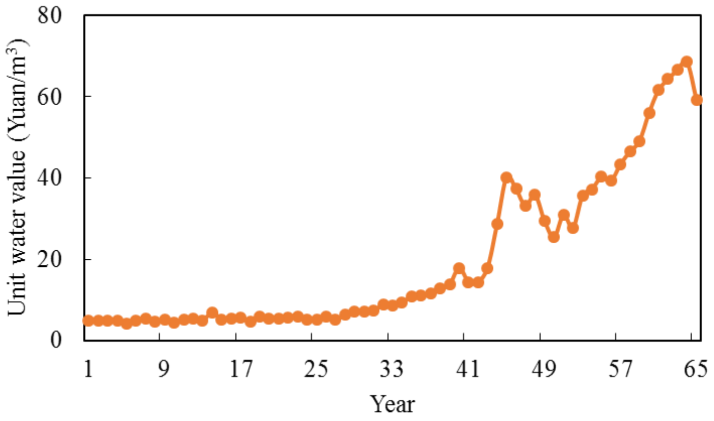

Figure S4 Unit water value for irrigation during 1951-2015

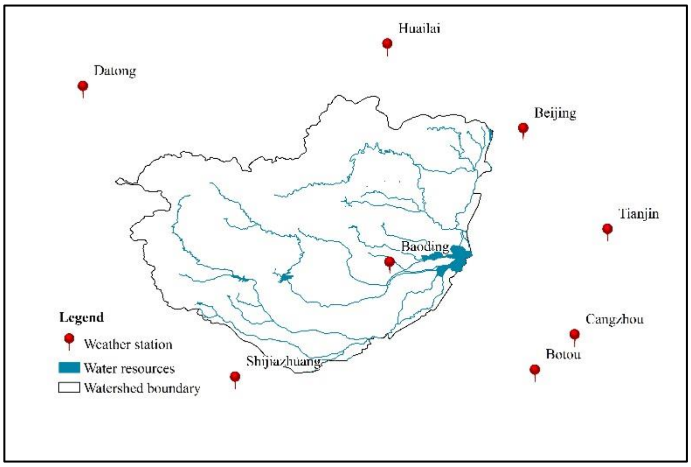

Figure S5 The location of weather stations
